# The state of child nutrition in Ethiopia: an umbrella review of systematic review and meta-analysis reports

**DOI:** 10.1186/s12887-020-02301-8

**Published:** 2020-08-26

**Authors:** Shimels Hussien Mohammed, Tesfa Dejenie Habtewold, Amanuel Godana Arero, Ahmad Esmaillzadeh

**Affiliations:** 1 Department of Community Nutrition, School of Nutritional Sciences and Dietetics, Tehran University of Medical Sciences, Tehran, Iran; 2 Department of Epidemiology, University Medical Center Groningen, University of Groningen, Groningen, The Netherlands; 3 Schoool of Medicine, Tehran University of Medical Sciences, Tehran, Iran; 4 Obesity and Eating Habits Research Center, Endocrinology and Metabolism Molecular Cellular Sciences Institute, Tehran University of Medical Sciences, Tehran, Iran; 5 Department of Community Nutrition, School of Nutritional Sciences and Dietetics, Tehran University of Medical Sciences, Tehran, Iran; 6 Food Security Research Center, Department of Community Nutrition, Isfahan University of Medical Sciences, Isfahan, Iran

**Keywords:** Malnutrition, Stunting, Wasting, Underweight, Complementary feeding, IYCF practices

## Abstract

**Background:**

Malnutrition remains to be a major public health problem in developing countries, particularly among children under-5 years of age children who are more vulnerable to both macro and micro-nutrient deficiencies. Various systematic review and meta-analysis (SRM) studies were done on nutritional statuses of children in Ethiopia, but no summary of the findings was done on the topic. Thus, this umbrella review was done to summarize the evidence from SRM studies on the magnitude and determinants of malnutrition and poor feeding practices among under-5 children in Ethiopia.

**Methods:**

PubMed, Embase, Scopus, Web of Sciences, Cochrane Database of Systematic Reviews, Database of Abstracts of Reviews of Effects, and Google Scholar were searched for SRM studies on magnitude and risk factors of malnutrition and child feeding practice indicators in Ethiopia. The methodological quality of the included studies was assessed using the Assessment of Multiple Systematic Reviews (AMSTAR) tool. The estimates of the included SRM studies on the prevalence and determinants of stunting, wasting, underweight, and poor child feeding practices were pooled and summarized with random-effects meta-analysis models.

**Result:**

We included nine SRM studies, containing a total of 214,458 under-5 children from 255 observation studies. The summary estimates of prevalence of stunting, underweight, and wasting were 42% (95%CI = 37–46%), 33% (95%CI = 27–39%), and 15% (95%CI = 12–19%), respectively. The proportion of children who met the recommendations for timely initiation of breastfeeding, exclusive breastfeeding during the first 6 months, and timely initiation of complementary feeding were 65, 60, and 62%, respectively. The proportion of children who met the recommendations for dietary diversity and meal frequency were 20, and 56%, respectively. Only 10% of children fulfilled the minimum criteria of acceptable diet. There was a strong relationship between poor feeding practices and the state of malnutrition, and both conditions were related to various health, socio-economic, and environmental factors.

**Conclusion:**

Child malnutrition and poor feeding practices are highly prevalent and of significant public health concern in Ethiopia. Only a few children are getting proper complementary feeding. Multi-sectoral efforts are needed to improve children’s feeding practices and reduce the high burden of malnutrition in the country.

## Background

Malnutrition remains to be a major public health concern in Ethiopia [1]. It is highly prevalent particularly among infants and young children, who are vulnerable to both macro and micro-nutrient deficiencies [2, 3]. Though malnutrition refers to both under- and over-nutrition conditions, the main malnutrition conditions of public health concern in Ethiopia are the ones related to under-nutrition, namely anemia, stunting, wasting, and underweight, the prevalence of each condition being above global averages [1, 4]. Malnutrition is of various negative consequences on the health and wellbeing of children. It has been linked to high child morbidity and mortality, poor cognitive, physical, and psychosocial development [5]. The effect of child malnutrition is not limited to only during childhood. It has also been linked to various chronic diseases during adulthood, including higher risks of obesity, cardiovascular morbidity, and mortality [6]. The economic consequences of malnutrition are also enormous. It negatively impacts work productivity and creates a great financial burden for the affected individual, the health system and the public at large [2, 6].

Malnutrition is a multifaceted condition, developing as a consequence of various dietary and non-dietary factors [7–11]. However, the most frequently mentioned and proximal determinants of child malnutrition are poor dietary quality, suboptimal child-caring practices and repeated childhood illnesses [2, 8, 12]. The World Health Organization (WHO) and United Nations Children’s Fund (UNICEF) have jointly outlined universal infant and young child feeding (IYCF) recommendations of high potential to reduce the burden of malnutrition and ensure optimal child health and nutritional status [12–14]. WHO and UNICEF recommend nations to make substantial progress in mainstreaming and implementing the IYCF recommendations. Early initiation of breastfeeding, exclusive breastfeeding during the first 6 months, continued breastfeeding, timely initiation of complementary food of optimal diversity and frequency, and micronutrients supplementation have taken centrality of the IYCF recommendations. Suboptimal IYCF practices are often associated with poor nutritional outcomes [13, 14]. The other non-dietary, but proximal, factors often linked to malnutrition are unhygienic environment and repeated infection, coupled with poor health care utilization [8–10, 12, 15]. The suboptimal practices in IYCF, hygiene, and health care utilization are in turn influenced by various underlying conditions like poor socioeconomic and educational statuses [2, 13].

A better understanding of the risks factors of malnutrition, particularly the locally responsible ones, is an important input for planning locally appropriate nutrition-enhancing measures [8]. Various systematic review and meta-analyses (SRM) studies have been reported on the magnitude and risk factors of child malnutrition and IYCF practices in Ethiopia [4, 16–23]. The main topics covered in the existing review works include stunting, wasting, underweight, dietary diversity and meal frequency. SRM reports have gained increasing recognition in policy-making processes. However, the SRM reports done on malnutrition and IYCF practices in Ethiopia were limited in their scope, including being focused on a specific malnutrition or IYCF aspect and falling short of providing a comprehensive picture of the situation. Besides, as the studies become more specific but increase in number, the information users (service providers or policymakers) would be overwhelmed with too many of them. Umbrella reviews facilitate evidence-based planning and decision making, by providing a ready summary of information of a broad topic area [24]. To the best of our knowledge, there is no previous comprehensive systematic review or umbrella review work that summarized the evidence from the existing SRM reports on the magnitude of malnutrition as well as IYCF practices in Ethiopia. Thus, we conducted this umbrella review of SRM studies done on the prevalence and determinants of malnutrition (stunting, wasting, underweight) and IYCF practices.

## Methods

This study was done following the methodology of umbrella review of SRM studies [24]. Umbrella review is a systematic synthesis of SRM reports on a specific research topic.

### Data source and literature search

Seven databases (PubMed, Embase, Scopus, Web of Sciences, Cochrane Database of Systematic Reviews, Database of Abstracts of Reviews of Effects (DARE), and Google Scholars) were searched for SRM studies on child malnutrition and IYCF practices in Ethiopia, published from January 2015 to August 15, 2019. The search for malnutrition studies was focused on the four more prevalent undernutrition conditions of public health priority in Ethiopia; i.e., anemia, stunting, underweight, wasting, and underweight [1, 4]. The search for IYCF practice studies was focused on the child feeding indicators recommended by WHO/UNICEF. They were (a) early initiation of breastfeeding, (b) exclusive breastfeeding during the first 6 months, (c) continued breastfeeding up to 2 years and beyond, (d) dietary diversity, and (e) meal frequency. Thus, we specifically searched for SRM studies that reported on the magnitudes and determinants the 4 malnutrition conditions and the IYCF practice indicators mentioned above. For each condition, key search terms were identified and used to develop search strategies. The key terms and phrases used for searching studies on malnutrition were ‘anemia’, ‘stunting’, ‘wasting’, ‘underweight’, ‘risk factor’, ‘predictor’, determinant’, ‘meta-analysis’, ‘systematic review’, and ‘review’. The key terms and phrases used for searching studies on IYCF practice were ‘early initiation of breastfeeding’, ‘within one-hour breastfeeding’, ‘exclusive breastfeeding’, ‘duration of breastfeeding’, ‘complementary feeding’, ‘timely initiation of complementary feeding’, ‘feeding practices’, ‘dietary diversity’, ‘dietary quality’, ‘dietary frequency’, ‘meal frequency’, ‘minimum acceptable diet’, and ‘IYCF practices’. The literature search was done by two reviewers independently, with discrepancy resolved by consensus. A sample of the literature search strategy, PubMed search strategy, developed using a combination of MeSH terms and free texts is presented as a supplementary file (see Additional file 1). In addition to the systematic database searching, article searching was done using the reference list of the included studies and the ‘cited by’ and ‘related articles’ function of PubMed.

### Study screening and selection

The search was restricted by language and period of publication. Only English language publications, done in the period 2015–2019, were eligible for inclusion. The time restriction was aimed to ensure the findings better reflect or relate to the current nutritional situation of the country. It was also for the magnitude and determinants of malnutrition might vary from time to time. For a study to be considered as systematic review or meta-analysis, it should have to meet the following predefined criteria: (a) presented a defined literature search strategy, (b) appraised included studies using a relevant tool, and (c) followed a standard approach in pooling studies and providing summary estimates. Studies were excluded due to any of the following reasons: (a) no report on the measures of interest for this study, (b) language other than English, and (c) narrative reviews, editorials, correspondence, abstracts, and methodological studies. When a study reported on more than one malnutrition conditions or IYCF practice indicators, all reports were extracted as long as they were reported following appropriate methods. The screening and selection of studies was conducted in two stages. First, title and abstract screening was done. Then, full-text reviewing was done.

### Data extraction

Data from the included studies were extracted using a standardized data abstraction form, developed in excel sheet. For each study, the following data were extracted: (a) identification data (first author’s last name and publication year), (b) type of malnutrition condition or IYCF practice indicator assessed, (c) measure of magnitude (prevalence for malnutrition, coverage or level of practice for IYCF indicators) or measure of association (odds ratio or relative risk) with 95% confidence intervals, (d) number of studies included, (e) total number of samples included, (f) risk factors (determinant or predictor reported) for the main outcome variable(s) in the study, (g) publication bias assessment methods and scores, (h) quality assessment methods and scores, (i) data synthesis methods (random or fixed-effects model), and (j) the main conclusion of the study. When a study provided two different estimates (i.e., one based on random-effects model and the other based on fixed-effects model) on the same outcome, we extracted the estimate from random-effects model if the associated between-studies heterogeneity (Higgin’s I^2^) [25] was > 50% and estimate from fixed-effects model if the associated heterogeneity was < 50%.

### Study quality and reliability assessment

The methodological quality of the included SRM studies was assessed using the Assessment of Multiple Systematic Reviews (AMSTAR) tool [26]. It consists of 11 questions that measure the quality of the approaches used for pooling the empirical studies included in the review and summarizing their estimates. The tool has been validated and frequently used for appraisal of the quality of SRM works. The quality scoring was done out of 11, with scores 8–11, 4–7, and < 3 indicating high, medium, and low qualities, respectively. The grading was done by two reviewers, with discrepancies resolved by discussion and consensus.

### Data synthesis

Both quantitative and qualitative approaches were used to summarize the estimates of the included studies. When two or more estimates were provided on the same topic, we presented the range of the estimates and also calculated a summary (pooled) estimate. The choice of the meta-analysis model was guided by the between-studies heterogeneity, which was assessed by Higgin’s I^2^-Statistics [25]. According to Higgins et al. I^2^ < 49%, 50–75, and > 75% represents low, moderate, and high levels of heterogeneity, respectively. We intended to pool the estimates with fixed-effects models if the level of heterogeneity was < 50%. However, there was a high level of between-studies heterogeneity. Thus, the pooled (summary) prevalence estimates were calculated with the DerSimonian-Laird random-effects model, which accounts for both within-study and between-studies variations [27]. We intended to assess publication bias by visual inspection of funnel plots, Begg’s rank or Egger’s regression tests, as appropriate. However, it was not possible to assess publication bias as there were inadequate numbers of studies, which under-power any of these methods. A minimum of 10 studies is needed to evaluate publication bias [28]. Stata version 15.0 software (StataCorp, TX USA) was used for the quantitative analyses. A summary list of determinants of malnutrition and poor IYCF practices was prepared.

### Ethical consideration

This study was done using data extracted from published studies. Thus, no study participants’ consent or ethical approval was needed.

## Result

### Literature search findings

**Fig. 1:**
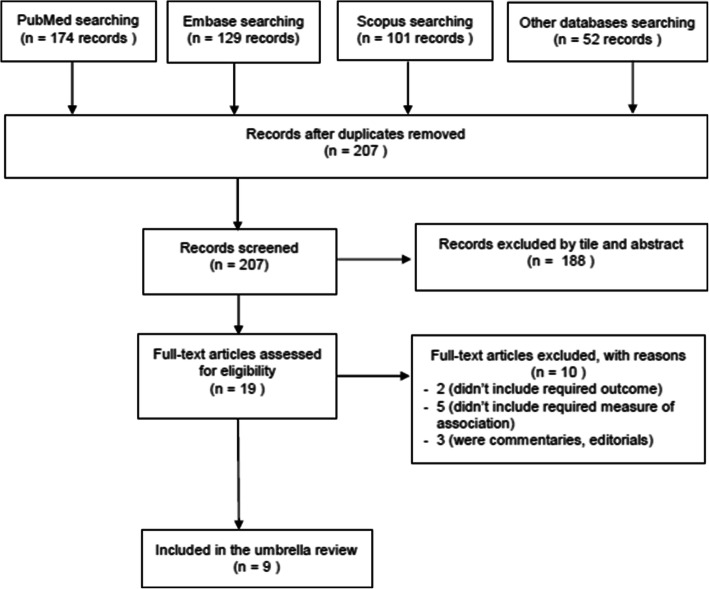
PRISMA flow chart of study screening and selection process

The database search provided a total of 207 articles, of which 19 were eligible for full-text review. The remaining studies which were not SRM studies were excluded because the objective of this study was to include only SRM studies on the topics of interest. After full text reviewing, 8 studies were found eligible for inclusion. Additionally, one article was found by hand searching of the reference lists of the included studies. Thus, a total of 9 studies [4, 16–23] were included in the current umbrella review. The study selection and screening process is shown in Fig. 1. We aimed to include anemia in this umbrella review, but no SRM report was found on it.

### Characteristics of included studies

All SRM studies included in this review were observational in design. They included a total of 255 studies, providing a total sample of 214,458 under-5 children. The number of studies per SRM ranged from 14 (lowest) [23] to 70 (highest) [21]. The sample size per meta-analysis ranged 13,531 (lowest) [23] to 55,000 (highest) [21]. All studies were published from 2017 to 2019. The specific malnutrition conditions assessed by the SRM studies were stunting, wasting, and underweight. Two meta-analyses were done on the prevalence and the determinants of stunting, underweight, and wasting [4, 16]. The specific IYCF practice indicators assessed were exclusive breastfeeding, early initiation of breastfeeding, timely initiation of complementary feeding, dietary diversity, meal frequency, and minimum acceptable diet. Seven studies were done on both the magnitude and the determinants of IYCF practices [17–23]. The overall characteristics of the included studies, including the topic they addressed, is shown in Table 1.

**Table 1:** General characteristics of included systematic review and meta-analyses studies

Author (year)	Study design	Age (months)	Included studies	Sample size	Main topic	Main measure	AMSTAR Quality
Abdulahi [4] (2017)	Survey	< 60	18	39,585	- Stunting - Underweight - Wasting	Prevalence	10
Abdurahman (2019) [17]	Survey	6–23	26	17, 383	- Timely initiation of breastfeeding - Minimum dietary diversity - Minimum meal frequency - Minimum acceptable diet	- Prevalence - Determinants	9
Alebel (2017) [18]	Survey	6–23	16	18,870	Timely initiation of breastfeeding	- Prevalence -Determinants	5
Habtewold (2018) [21]	Survey	6–23	70	55,000	- Timely initiation of breastfeeding - Exclusive breastfeeding - Timely initiation of breastfeeding	- Prevalence - Determinants	10
Temesgen (2019) [23]	Survey	6–23	14	13,531	Minimum dietary diversity	- Prevalence - Determinants	8
Abate (2019) [16]	Survey	< 60	23	18,172	Stunting	Determinants	5
Alebel (2018) [19]	Survey	6–23	32	23,543	Exclusive breastfeeding	Prevalence	5
Habtewold (2019) [22]	Survey	6–23	25	31,066	Timely initiation of breastfeeding	Determinants	10
Habtewold (2019) [20]	Survey	6–23	31	14,691	Exclusive breastfeeding	Determinants	10

*AMSTAR* Assessment of Multiple Systematic Reviews

### Methodological quality of included studies

Table 2 shows the methodological quality of the included studies, evaluated using the AMSTAR tool for assessment of the methodological quality of SRM studies [26]. The quality scoring was done out of 11 points and ranged from 5 to 10, with a mean score of 7.8 points, indicating an overall moderate quality. The AMSTAR criteria more frequently satisfied across the studies were the ones about the assessment of publication bias and disclosure of conflict of interest. The AMSTAR criteria less frequently satisfied were the ones about inclusion and exclusion of studies and priori design.

**Table 2:** Methodological quality of the included studies based on the AMSTAR tool

Author, year	Q1	Q2	Q3	Q4	Q5	Q6	Q7	Q8	Q9	Q10	Q11	Total
Habtewold (2018) [21]	Yes	Yes	Yes	Yes	Yes	Yes	Yes	No	Yes	Yes	Yes	10
Abdurahman (2019) [17]	Yes	Yes	Yes	Yes	No	Yes	Yes	No	Yes	Yes	Yes	9
Temesgen (2019) [23]	No	Yes	Yes	Yes	No	Yes	Yes	No	Yes	Yes	Yes	8
Alebel (2017) [18]	No	Yes	No	No	No	Yes	Yes	No	Yes	Yes	Yes	5
Abdulahi(2017) [4]	Yes	Yes	Yes	Yes	No	Yes	Yes	No	Yes	Yes	Yes	10
Alebel (2018) [19]	No	Yes	No	No	No	Yes	Yes	No	Yes	Yes	Yes	5
Habtewold (2019) [22]	Yes	Yes	Yes	Yes	Yes	Yes	Yes	No	Yes	Yes	Yes	10
Habtewold (2019) [20]	Yes	Yes	Yes	Yes	Yes	Yes	Yes	No	Yes	Yes	Yes	10
Abate (2019) [16]	No	Yes	No	Yes	No	No	Yes	No	No	Yes	Yes	5

*AMSTAR* Assessment of Multiple Systematic Reviews

Q1: A priori design; Q2: Duplicate study selection and data extraction; Q3: Search comprehensiveness; Q4: Inclusion of grey literature; Q5: Included and excluded studies provided; Q6: Characteristics of the included studies provided; Q7: Scientific quality of the primary studies assessed and documented; Q8: Scientific quality of included studies used appropriately in formulating conclusions; Q9: Appropriateness of methods used to combine studies’ findings; Q10: Likelihood of publication bias was assessed; Q11: Conflict of interest – potential sources of support were clearly acknowledged in both the systematic review and the included studies

### Magnitude and determinants of malnutrition

The SRM studies on the magnitude and determinants of malnutrition included a total of 41 cross-sectional studies, covering a total sample of 57,757 under-5 children. The summary pooled prevalence of stunting, as defined by WHO height-for-age Z-scores below 2 standard deviations (SD) from the median of the reference population, was 42% (95%CI = 37–46%). The summary pooled prevalence of underweight, as defined by WHO weight-for-age Z-scores below 2SD from the median of the reference population, was 33% (95%CI = 27–39%). The summary pooled prevalence of wasting, as defined by WHO weight-for-height Z-scores below 2SD from the median of the reference population, was 15% (95%CI = 12–19%). The summary estimates of the prevalence of malnutrition are shown in Table 3.

**Table 3:** Summary of the prevalence of malnutrition and indicators of child feeding practices

Variable or indicator	Reference	No. of Studies	Sample size	Reported prevalence		Summary prevalence^a^	
				P(95%CI)	I^**2**^(%)	P(95%CI)	I^**2**^(%)
Stunting	Abdulahi (2017) [4]	18	39,585	42 (37–46)	98.5	42 (37–46)	98.5
Underweight	Abdulahi (2017) [4]	17	28,169	33 (27–39)	99.0	33 (27–39)	99.0
Wasting	Abdulahi (2017) [4]	16	30,658	15 (12–19)	98.9	15 (12–19)	98.9
Timely breastfeeding initiation	Habtewold (2018) [21]	45	47,858	67 (62–71)	99.0	65 (65–66)	1.9
	Alebel (2017) [18]	16	18,870	61 (51–72)	99.4		
Exclusive breastfeeding	Habtewold (2018) [21]	40	25,816	60 (56–65)	98.0	60 (59–60)	0.0
	Alebel (2018) [19]	32	23,543	59 (54–65)	98.7		
Timely complementary feeding initiation	Habtewold (2018) [21]	21	55,000	63 (57–68)	97.0	62 (61–63)	4.1
	Abdurahman (2019) [17]	14	17,383	61 (52–70)	98.5		
Minimum dietary diversity	Abdurahman (2019) [17]	19	17, 383	18 (11–25)	99.5	20 (19–21)	2.8
	Temesgen (2019) [23]	14	13,531	23 (18–29)	98.8		
Minimum meal frequency	Abdurahman (2019) [17]	14	17, 383	56 (45–66)	99.2	56 (45–66)	99.2
Minimum acceptable diet	Abdurahman (2019) [17]	8	17, 383	10 (07–14)	91.5	10 (07–14)	91.5

*P* Prevalence, *CI* Confidence interval

^a^Calculated with random-effects meta-analysis model

The multi-dimensional factors, i.e. dietary and non-dietary factors, found linked to any of the three malnutrition conditions are shown in Table 4. Of these, the most frequently mentioned dietary factors founded linked to high risk of malnutrition (stunting, underweight, and wasting) were late initiation of breastfeeding, non-exclusive breastfeeding during the first 6 months, late initiation of complementary feeding, and low diversity and frequency of complementary feeding. Environmental factors found often associated with a high risk of malnutrition were an unimproved household water source, unimproved household toilet facility, and rural place of residence. Health factors found often associated with a high risk of malnutrition were childhood infection, home delivery, lack of immunization, family planning, antenatal and postnatal care, and poor utilization of micronutrient supplements like iron, vitamin A, and prophylaxis medications like deworming. There was significant variation in the magnitude of malnutrition by children’s sex and age; such that, there was a significant difference in the prevalence of stunting, wasting, and underweight by age and sex.

**Table 4:** Summary of risk factors of malnutrition and poor IYCF practices

Outcome	Risk factors	
Malnutrition	Dietary/Feeding [4, 16]	Poor breastfeeding and complementary feeding
		Food insecurity
	Health [4, 16]	Lack of antenatal care
		Lack of postnatal care
		Deworming
		Vitamin A supplementation
		Immunization
		Counseling
		Infection
		Place of delivery
	Sociodemographic [4, 16]	Child sex
		Child age
		Maternal education status
		Wealth (income)
		Family size
		Media exposure
	Hygiene [4, 16]	Type water source
		Type of toilet facility
	Environmental [4, 16]	Place of residence
IYCF practices	Health [17–23]	Lack of antenatal care
		Lack of postnatal care
		Place of delivery
	Sociodemographic [17–23]	Child sex
		Child age
		Maternal education status
		Wealth (income)
		Family size
		Media exposure
		Paternal involvement
		IYCF knowledge
		Breastfeeding experience
	Environmental [17–23]	Place of residence

*IYCF* Infant and young child feeding

### Magnitude and determinants of IYCF practice indicators

Seven SRM studies were done on the magnitude and determinants of suboptimal IYCF practice indicators. The specific IYCF indicators assessed were early initiation of breastfeeding, exclusive breastfeeding, timely initiation of complementary feeding, minimum dietary diversity, minimum meal frequency, and minimum acceptable diet. No SRM report was found on the duration of breastfeeding. The reported estimate of the level of early initiation of breastfeeding ranged from 61% (95%CI = 51–72%) to 67% (95%CI = 62–71%) and the pooled prevalence (calculated summary) estimate was 65% (65–55%); such that, two-thirds of children were fed with breast milk within the first 1 h after birth. The reported estimate of the level of exclusive breastfeeding ranged from 59% (95%CI = 54–65%) to 60% (95%CI = 56–65%) and the pooled prevalence (calculated summary) estimate was 60% (95%CI = 59–60%). The reported estimate of the level of timely initiation of complementary feeding ranged from 61% (95%CI = 52–70%) to 63% (95%CI = 57–68%) and the pooled prevalence (calculated summary) estimate was 62% (95%CI = 61–63%). The reported estimate of the proportion of children who met the minimum dietary diversity ranged from 18% (95%CI = 11–25%) to 23% (95%CI = 18–29%) and the pooled (calculated summary) estimate was 20% (95%CI = 19–21%). The summary estimates of the proportion of children who met the minimum meal frequency and the minimum acceptable diet were 56.0% (95%CI = 45–66%) and 10.0% (95%CI = 7–14%), respectively. Table 3 shows the reported and calculated (pooled) summary estimates of IYCF practices.

Seven SRM studies [17–23] examined factors associated with sub-optimal IYCF practices and reported a number of health, sociodemographic, and environmental factors. Home delivery (i.e., instead of intuitional delivery), not attending antenatal care, postnatal care, and nutritional counseling services were the main health-related factors often found linked to sub-optimal IYCF practices. Low caregivers’ educational status, poor household socioeconomic status (low wealth category), low caregivers’ media exposure, paternal involvement in child’s care, household family size, and maternal breastfeeding experience were the main sociodemographic found linked to poor IYCF practices. Like the case of malnutrition, there was also significant variation in IYCF practices by children’s sex and age. Rural residence was the main environmental or household factor found linked to poor IYCF practices.

## Discussion

The last decade has seen a significant rise in the number of SRM reports on various nutritional topics. SRM studies represent a high body of evidence for decision making in health/nutrition programs. However, it would be overwhelming for the information user when the number of specific reviews increases [24]. Thus, this umbrella review was conducted to summarize the existing SRM studies on nutritional status and feeding practices of under-5 children in Ethiopia and found that stunting, underweight and wasting were highly prevalent and of significant public health concern in the country. Complementary feeding practices were largely sub-optimal in most children, with only a few of them benefiting from proper quality of complementary feeding. Both the high magnitude of malnutrition and the suboptimal IYCF practices were linked to various socio-economic, health, and environmental factors.

This review found clear evidence that malnutrition is still a major public health problem among under-5 children in Ethiopia. The prevalence of each of stunting, underweight and wasting was high and above the acceptable international standards. Stunting was the most prevalent of the three conditions. With two-fifths of under-5 children being stunted, Ethiopia bears one of the highest global stunting burdens. In 2018, the prevalence of stunting was estimated to be 22% globally, 24% in developing countries, and 6% in developed countries [29]. Stunting reflects not only linear growth failure but also the child’s overall poor health and wellbeing. Most growth faltering occurs during the first 2 years and is often irreversible once happened [3]. WHO classifies stunting prevalence above 40% as a severe public health problem [29, 30]. Thus, the case of stunting in Ethiopia warrants serious public health attention. The levels of underweight and wasting in the country were also higher than the corresponding global and African averages. In 2018, the global prevalence of wasting was 7% [31]. WHO recommends that the proportion of wasted children should not exceed 5% and a value above 10% is considered as a severe public health problem [30]. Based on this reference, the case of wasting in Ethiopia (15%) is also of a significant public health concern.

This study also found a high level of poor child feeding practices in Ethiopia. Only a few children were fed with an optimal diet, appropriate in both diversity and frequency. To reduce the global burden of malnutrition, WHO has outlined essential IYCF recommendations [12, 13, 32]. The IYCF recommendations are designed specifically for children under 24 months of age and provide universal guidance for optimal breast and complementary feeding practices. The optimal breastfeeding recommendations include starting breastfeeding within the first 1 h after birth, exclusive breastfeeding during the first 6 months of age, and continued breastfeeding up to 2 years and beyond [12, 13, 32]. Breastmilk alone could not satisfy the nutrient demand of a child after 6 months of age [13]. Thus, the child needs to get appropriate complementary food, starting from 6 months of age. An appropriate complementary food should be composed of at least four food items and the frequency of complementary food feeding should be at least three times a day for breastfeeding children and at least four times a day for non-breastfeeding children [12, 13, 32]. In this study, it was found that the minimum dietary diversity and the minimum meal frequency criteria were not satisfied for the majority of children in Ethiopia. Only 10% of children fulfilled the minimum acceptable diet quality. This is of a great concern as inadequate complementary feeding leads to macro- and micro-nutrient deficiency state, the consequences of which is often serious during childhood and might extend to even adulthood [13]. The problem of poor complementary feeding is not limited to Ethiopia. A previous review has shown that only too few children are benefitting from proper complementary feeding globally [13, 14]. Compared to complementary feeding, breastfeeding was better practiced in Ethiopia. Most children started breastfeeding early and were exclusively breastfed during the first 6 months. However, this does not mean that there was optimal breastfeeding practice in Ethiopia. Rather, efforts need to be made to ensure all children start breastfeeding early and be breastfed exclusively during the first 6 months after birth [13, 14].

Both malnutrition and poor IYCF practices were found linked to various sociodemographic, health, and environmental factors. The finding was consistent with the multifactorial nature of malnutrition [13] and the reports of previous studies done in Ethiopia as well as other developing countries [11, 33–35]. According to the UNICEF conceptual framework of causation of malnutrition, the risk factors of malnutrition could be categorized as immediate, underlying, and basic determinants [8]. The main immediate risk factors are inadequate food intake and infection. The main underlying factors are food insecurity, poor childcare, and unhygienic practices, coupled with poor health care utilization. Poverty and illiteracy are the most frequently mentioned basic determinants of malnutrition [8, 36, 37].

Our findings have important policy and research implications. The information could serve as an input for decision making, resource allocation, and design of interventions to improved IYCF practices as well as reduce the burden of poor child nutritional outcomes in Ethiopia. Since long, prevention and control of malnutrition has been a priority agenda in Ethiopia [1, 38]. However, the rate of reduction has been slow and frustrating [1]. WHO recommends a 40% reduction in the proportion of stunted children by 2022 from the figure in 2010 [29]. With the current less promising rate of reduction, it seems unlikely for Ethiopia to meet the 40% reduction goal unless a concerted effort is done in the remaining years. To that end, it is important for Ethiopia to accelerate the implementation of both nutrition-specific and nutrition-sensitive measures [39]. As malnutrition is a multifactorial condition, it is essential to coordinate comprehensive and multi-sectorial interventions across all sectors with a stake on nutrition. Thus, the provision of all of the essential nutrition interventions recommended by the WHO [12] like child immunization, micronutrient supplementation (like timely vitamin A supplementation), deworming medications, growth monitoring and promotion, water, sanitation, and hygiene need also be strengthened together with improving IYCF practices. Allocating adequate resource, prioritizing the most vulnerable population groups, and periodic performance evaluation are also important to achieve the goal of malnutrition reduction in Ethiopia and other developing countries.

To the best our knowledge, no comprehensive assessment (umbrella review) has been done on the state of child nutrition in Ethiopia, albeit various empirical and specific SRM studies are available. The study has some important limitations worth mentioning to the reader. All the studies included in this study were done using cross-sectionally conducted studies. Thus, this review also shares the limitations of observational research design; such that a cause-effect relationship could not be inferred on any of the estimates provided. There was high heterogeneity among the included studies, which might have biased the summary estimates. Not all malnutrition forms and IYCF indicators are covered in this work due to the lack of SRM reports on issues like anemia, vitamin A deficiency, and iodine deficiency. Further umbrella reviews are needed when more SRS reports become available in the future.

## Conclusion

Stunting, underweight, and wasting are highly prevalent among infants and young children in Ethiopia. Most IYCF recommendations, particularly those related to diversity of diet and frequency of feeding, are poorly practiced. Only too few children benefit from proper complementary feeding practices. Both malnutrition and poor IYCF practices are linked to various multi-dimensional factors. The high magnitude of malnutrition as well as the suboptimal complementary feeding practices warrant serious public health concern and urgent response. Enhancing both nutrition-specific and nutrition-sensitive measures through a coordinated, integrated and multi-sectoral approach stands worth considering to improve IYCF practices and consequently reduce the burden of malnutrition in Ethiopia.

## Supplementary information

**Additional file 1.** PubMed Search Strategy.

## Data Availability

All data are included within the manuscript.

## Abbreviations

AMSTAR: Assessment of multiple systematic reviews
CI: Confidence interval
DARE: Database of abstracts of reviews of effects
IYCF: Infant and young child feeding practice
MeSH: Medical subjects headings
UNICEF: United Nations Children’s Fund
WHO: World health organization
SRM: Systematic review and meta-analysis

## References

[1] Central Statistical Agency [Ethiopia] and ICF International (2016). Ethiopia Demographic and Health Survey.

[2] Bhutta ZA, Das JK, Rizvi A (2013). Evidence-based interventions for improvement of maternal and child nutrition: what can be done and at what cost?. Lancet.

[3] Victora CG, de Onis M, Hallal PC (2010). Worldwide timing of growth faltering: revisiting implications for interventions. Pediatrics.

[4] Abdulahi A, Shab-Bidar S, Rezaei S (2017). Nutritional status of under five children in Ethiopia: a systematic review and meta-analysis. Ethiop J Health Sci.

[5] Smith L, Haddad L (2014). Reducing child Undernutrition: past drivers and priorities for the post-MDG era. IDS Working Papers.

[6] Alderman H, Hoddinott J, Kinsey B (2006). Long term consequences of early childhood malnutrition. Oxf Econ Pap.

[7] Danaei G, Andrews KG, Sudfeld CR (2016). Risk factors for childhood Stunting in 137 developing countries: a comparative risk assessment analysis at global, regional, and country levels. PLoS Med.

[8] United Nations Children’s Fund (1991). Strategy for improved nutrition of children and women in developing countries. United Nations Children’s Fund. Indian J Pediatr.

[9] Mohammed SH, Habtewold TD, Esmaillzadeh A (2019). Household, maternal, and child related determinants of hemoglobin levels of Ethiopian children: hierarchical regression analysis. BMC Pediatr.

[10] Mohammed SH, Habtewold TD, Tegegne BS (2019). Dietary and non-dietary determinants of linear growth status of infants and young children in Ethiopia: hierarchical regression analysis. PLoS One.

[11] Derso T, Tariku A, Biks GA (2017). Stunting, wasting and associated factors among children aged 6-24 months in Dabat health and demographic surveillance system site: a community based cross-sectional study in Ethiopia. BMC Pediatr.

[12] World Health Organization (2013). Essential nutrition actions: improving maternal N, infant and young child health and nutrition.

[13] White JM, Bégin F, Kumapley R, Murray C, Krasevec J (2017). Complementary feeding practices: current global and regional estimates. Maternal Child Nutr.

[14] Abeshu MA, Lelisa A, Geleta B (2016). Complementary feeding: review of recommendations, feeding practices, and adequacy of homemade complementary food preparations in developing countries - lessons from Ethiopia. Front Nutr.

[15] Vilcins D, Sly PD, Jagals P (2018). Environmental risk factors associated with child Stunting: a systematic review of the literature. Ann Global Health.

[16] Abate KH, Belachew T (2019). Chronic malnutrition among under five children of Ethiopia may not be economic. A systematic review and meta-analysis. Ethiop J Health Sci.

[17] Abdurahman AA, Chaka EE, Bule MH (2019). Magnitude and determinants of complementary feeding practices in Ethiopia: a systematic review and meta-analysis. Heliyon.

[18] Alebel A, Dejenu G, Mullu G (2017). Timely initiation of breastfeeding and its association with birth place in Ethiopia: a systematic review and meta-analysis. Int Breastfeed J.

[19] Alebel A, Tesma C, Temesgen B (2018). Exclusive breastfeeding practice in Ethiopia and its association with antenatal care and institutional delivery: a systematic review and meta-analysis. Int Breastfeed J.

[20] Habtewold TD, Endalamaw A, Mohammed SH, et al. Multidimensional factors predicting exclusive breastfeeding in Ethiopia: evidence from a meta-analysis of studies in the past 10 years. medRxiv. 2019:19002857. 10.1101/19002857. 10.1007/s10995-020-03059-2 33389586

[21] Habtewold TD, Mohammed SH, Endalamaw A (2019). Breast and complementary feeding in Ethiopia: new national evidence from systematic review and meta-analyses of studies in the past 10 years. Eur J Nutr.

[22] Habtewold TD, Mohammed SH, Endalamaw A, et al. Proximal and distal factors predicting timely initiation of breastfeeding in Ethiopia: a systematic review and meta-analysis. medRxiv. 2019:19000497. 10.1101/19000497.

[23] Temesgen H, Negesse A, Woyraw W (2018). Dietary diversity feeding practice and its associated factors among children age 6-23 months in Ethiopia from 2011 up to 2018: a systematic review and meta-analysis. Ital J Pediatr.

[24] Aromataris E, Fernandez R, Godfrey CM, Holly C, Khalil H, Tungpunkom P (2015). Summarizing systematic reviews: methodological development, conduct and reporting of an umbrella review approach. Int J Evidence-Based Healthcare.

[25] Higgins JP, Thompson SG (2002). Quantifying heterogeneity in a meta-analysis. Stat Med.

[26] Shea BJ, Grimshaw JM, Wells GA (2007). Development of AMSTAR: a measurement tool to assess the methodological quality of systematic reviews. BMC Med Res Methodol.

[27] DerSimonian R, Laird N (1986). Meta-analysis in clinical trials. Control Clin Trials.

[28] Valentine JC, Pigott TD, Rothstein HR (2010). How many studies do you need? A primer on statistical power for meta-analysis. J Educ Behav Stat.

[29] De Onis M, Blössner M, Borghi E (2012). Prevalence and trends of stunting among pre-school children, 1990–2020. Public Health Nutr.

[30] Akombi BJ, Agho KE, Hall JJ (2017). Stunting, wasting and underweight in Sub-Saharan Africa: a systematic review. Int J Environ Res Public Health.

[31] United Nations Children’s Fund (2019). Levels and trends in child malnutrition.

[32] World Health Organization (2010). Indicators for assessing infant and young child feeding practices part III country profiles.

[33] Hemalatha R, Radhakrishna KV, Kumar BN (2018). Undernutrition in children & critical windows of opportunity in Indian context. Indian J Med Res.

[34] Lutter CK, Daelmans BM, de Onis M (2011). Undernutrition, poor feeding practices, and low coverage of key nutrition interventions. Pediatrics.

[35] Mohammed SH, Larijani B, Esmaillzadeh A (2019). Concurrent anemia and stunting in young children: prevalence, dietary and non-dietary associated factors. Nutr J.

[36] Mohammed SH, Muhammad F, Pakzad R (2019). Socioeconomic inequality in stunting among under-5 children in Ethiopia: a decomposition analysis. BMC Res Notes.

[37] Steyn NP, Walker AR (2000). Nutritional status and food security in sub-Saharan Africa: predictions for 2020. Asia Pac J Clin Nutr.

[38] Lemma F, Matji J (2013). Delivery platforms for sustained nutrition in Ethiopia. Lancet.

[39] Kraemer K, Cordaro J, Fanzo J, et al. Nutrition-Specific and Nutrition-Sensitive Interventions. Good Nutrition: Perspectives for the 21st Century. Basilea: Karger Publishers; 2016. p. 276–88.

