# Supplementary material for: The state of child nutrition in Ethiopia: an umbrella review of systematic review and meta-analysis reports
Source: BMC Pediatr. 2020 Aug 26;20:404. doi: 10.1186/s12887-020-02301-8 (PMC7448348; doi:10.1186/s12887-020-02301-8)
Supplement: Supplementary file 1 — Additional file 1. PubMed Search Strategy. [file 12887_2020_2301_MOESM1_ESM.pdf]

Additional file 1: PubMed Search Strategy

Search	Query	Hits*
#1	(((((((((((((((((Stunting[MeSH Terms]) OR Wasting[MeSH Terms]) OR Underweight[MeSH Terms] OR Stunting[Title/Abstract] OR Underweight[Title/Abstract]) OR Under-weight[Title/Abstract]) OR Wasting[Title/Abstract]) OR Breastfeeding[MeSH Terms]) OR Complementary feeding[MeSH Terms]) OR Child feeding practice[MeSH Terms]) OR Dietary diversity[MeSH Terms]) OR Meal frequency[MeSH Terms]) OR Breastfeeding[Title/Abstract]) OR Complementary feeding[Title/Abstract]) OR Child feeding practice[Title/Abstract]) OR Dietary diversity[Title/Abstract]) OR Meal frequency[Title/Abstract])	1049827
#2	((((((((Risk factor[MeSH Terms]) OR Prevalence[MeSH Terms]) OR Coverage[MeSH Terms]) OR Risk factor[Title/Abstract]) OR Prevalence[Title/Abstract]) OR Coverage[Title/Abstract]) OR Determinants[Title/Abstract]) OR Predictors[Title/Abstract])	295170
#3	((((Review[MeSH Terms]) OR Review[Title/Abstract]) OR Systematic Review[Title/Abstract]) OR Meta-analysis[Title/Abstract])	804616
#4	#1 AND #2 AND #3	207

**= Date of search: August 15, 2019*
